# Subchondral pre-solidified chitosan/blood implants elicit reproducible early osteochondral wound-repair responses including neutrophil and stromal cell chemotaxis, bone resorption and repair, enhanced repair tissue integration and delayed matrix deposition

**DOI:** 10.1186/1471-2474-14-27

**Published:** 2013-01-16

**Authors:** Charles-Hubert Lafantaisie-Favreau, Jessica Guzmán-Morales, Jun Sun, Gaoping Chen, Adam Harris, Thomas D Smith, Alberto Carli, Janet Henderson, William D Stanish, Caroline D Hoemann

**Affiliations:** 1 Institute of Biomedical Engineering, École Polytechnique, C.P. 6079 succ. Centre-Ville, Montréal, QC, H3C 3A7, Canada; 2 Department of Chemical Engineering, École Polytechnique, C.P. 6079 succ. Centre-Ville, Montréal, QC, H3C 3A7, Canada; 3 Biosyntech/Piramal HealthCare Canada Inc, 475 Armand Frappier, Laval, QC, H7V 4B3, Canada; 4 Current address: Comparative Orthopaedic Research Lab, Department of Clinical Studies, University of Guelph, 50 McGilvray, Lane Guelph, ON, N1G 2W1, Canada; 5 The Research Institute of the McGill University Health Centre, Montreal General Hospital, 1650 Cedar Ave, Montréal, QC, H3G 1A4, Canada; 6 Orthopaedic and Sport Medicine Clinic of Nova Scotia, Dalhousie University, 5595 Fenwick St., Suite 311, Halifax, NS, B3H 4M2, Canada

**Keywords:** Cartilage repair, Bone marrow, Chitosan, Osteoclast, Neutrophil, Collagen, Marrow stimulation, Bone remodeling, Mesenchymal stromal cell, Micro-computed tomography

## Abstract

**Background:**

In this study we evaluated a novel approach to guide the bone marrow-driven articular cartilage repair response in skeletally aged rabbits. We hypothesized that dispersed chitosan particles implanted close to the bone marrow degrade *in situ* in a molecular mass-dependent manner, and attract more stromal cells to the site in aged rabbits compared to the blood clot in untreated controls.

**Methods:**

Three microdrill hole defects, 1.4 mm diameter and 2 mm deep, were created in both knee trochlea of 30 month-old New Zealand White rabbits. Each of 3 isotonic chitosan solutions (150, 40, 10 kDa, 80% degree of deaceylation, with fluorescent chitosan tracer) was mixed with autologous rabbit whole blood, clotted with Tissue Factor to form cylindrical implants, and press-fit in drill holes in the left knee while contralateral holes received Tissue Factor or no treatment. At day 1 or day 21 post-operative, defects were analyzed by micro-computed tomography, histomorphometry and stereology for bone and soft tissue repair.

**Results:**

All 3 implants filled the top of defects at day 1 and were partly degraded *in situ* at 21 days post-operative. All implants attracted neutrophils, osteoclasts and abundant bone marrow-derived stromal cells, stimulated bone resorption followed by new woven bone repair (bone remodeling) and promoted repair tissue-bone integration. 150 kDa chitosan implant was less degraded, and elicited more apoptotic neutrophils and bone resorption than 10 kDa chitosan implant. Drilled controls elicited a poorly integrated fibrous or fibrocartilaginous tissue.

**Conclusions:**

Pre-solidified implants elicit stromal cells and vigorous bone plate remodeling through a phase involving neutrophil chemotaxis. Pre-solidified chitosan implants are tunable by molecular mass, and could be beneficial for augmented marrow stimulation therapy if the recruited stromal cells can progress to bone and cartilage repair.

## Background

Marrow stimulation is a cartilage repair technique in which bone channels are created at the base of the debrided cartilage lesion, to permit migration of bone marrow-derived repair cells into the lesion. Marrow stimulation techniques most often elicit fibrous or fibrocartilaginous tissue [1] that breaks down with time, with a documented ~25% failure rate at 5 years post-operative [2]. Therefore, implants that elicit a more hyaline repair tissue quality from marrow stimulation defects are currently under intense research.

In designing new treatments to regenerate articular cartilage, biomaterials that do not need to be seeded with cultured cells have the advantage of being clinically practical and cost-efficient. However, to be effective, biomaterial-only implants must be able to guide mesenchymal stem cells with chondrogenic potential from the bone marrow to above the osteochondral junction and into the cartilage lesion. Biomaterials have previously been successfully implanted into osteochondral defects, but reproducible long-term clinical benefits have yet to be demonstrated. A tubular implant composed of electrospun poly(D,L-lactide-co-glycolide) (PLG) inserted in large (5 mm wide, 5 mm deep) osteochondral defects in a rabbit model generated good cartilage repair in most defects at 24 weeks post-operative [3]. Subchondral implants made of PLG copolymer, calcium sulfate, polyglycolide fibers and surfactant have demonstrated clinical benefit, with complete pain alleviation and resumption of functional activity after 24 months of continued rehabilitation in a case study [4]; although other studies reported that PLG persists and inhibits subchondral bone regeneration 6 to 12 months post-operative [5,6]. In a rabbit model, filling the drill hole with a chemically cross-linked chitosan hydrogel was previously shown to block cell infiltration and bone repair [7]. Altogether, these data suggest that the porosity and degradation kinetics of biomaterials implanted in subchondral bone are key elements in the induction of reproducible osteochondral repair.

It was previously shown that hybrid chitosan/blood implants solidified *in situ* over the surface of microfracture or microdrill defects elicit a more hyaline repair compared to marrow stimulation alone [8,9]. The chitosan used to generate these implants was biodegradable with ~80% degree of deacetylation (DDA) and a molecular weight of ~100-250 kDa. The mechanisms of action were shown in a rabbit model to implicate the sequential attraction of neutrophils, osteoclasts and blood vessels, along with enhanced subchondral bone remodeling, repair tissue integration and delayed chondrogenesis in repairing microdrill holes [9-12]. The cartilage repair response, however, was attenuated in aged rabbits [10], which parallels the reduced efficacy of marrow stimulation therapies in patients over 40 years old [13,14]. A new approach is thus needed to reproducibly elicit hyaline repair in older patients treated by microfracture. Rabbits are widely used as an *in vivo* cartilage repair model, which provides an abundant base of literature for data comparison, and the use of aged rabbits in a cartilage repair model could be useful in translating new implants for augmented microfracture to treatments for older subjects.

We hypothesized that chitosan/blood implants placed directly in the marrow stimulation subchondral bone channel of skeletally aged rabbits could attract more bone marrow stromal cells than drilled control defects. We also hypothesized that subchondral chitosan/blood implants would be cleared with kinetics similar to a blood clot and, compared to drilled control defects, would attract more wound repair cells (neutrophils and marrow-derived stromal cells) in a molecular weight-dependent manner. Implants were delivered to drilled subchondral defects to avoid bone compaction and osteocyte death induced by microfracture [15]. In addition, our study was designed to track tissue reactions of three implant formulations with distinct chitosan molecular weight in 3 drill holes per knee trochlea. The generation of three structurally distinct fluorescent chitosan tracers with the same rhodamine derivatization level (1 tag per 200 monomers) [16] is an innovation that permitted comparative monitoring of the chitosan particle fate after 1-day and 3-weeks of *in vivo* repair. This formulation screening experiment will thus allow the identification of the optimal chitosan molecular weight to be used for subchondral implantation in subsequent studies.

## Methods

### Study design

Three osteochondral drill holes were created bilaterally in the knee trochlea of 30-month old New Zealand White rabbits. In one knee, the 3 holes were press-fit with 3 distinct pre-solidified chitosan-NaCl/blood implants containing chitosan (either high, low or ultra-low molecular weight, inserted in the proximal, middle, and distal holes, respectively). The 3 drill holes created in the contralateral control trochlea were left to bleed as surgical controls or each drill hole treated with the same dose of Tissue Factor used to pre-solidify the chitosan/blood implants. Individual drill holes (30 total holes) and associated repair tissues were analyzed after 1 day (N=1 rabbits) or 21 days (N=4 rabbits) post-operatively by micro-computed tomography to analyze residual bone defect size, and by histology and histomorphometry to assess implant residency, cell recruitment, matrix deposition, evidence of new bone formation, and repair tissue integration.

### Materials and chitosan characterization

High molecular weight chitosans (80% - 82% degree of deacetylation, DDA, endotoxins <500 EU/g, <0.2% protein, <5ppm heavy metals, <0.2% ash) were obtained from BioSyntech (Laval, QC, Canada, now Piramal Healthcare), from which 81.9% DDA chitosan was depolymerized by nitrous acid as previously described [17] to a targeted number-average molecular weight (*M*_n_) of ~40 kDa, and ~10 kDa, respectively (Table 1, 40K and 10K, and 150K). Nitrous acid cleaves the β–O-(1–4) glycosidic linkages without affecting the N-acetyl groups [18], and the DDA of the depolymerized chitosan was assumed to be identical to the DDA of the parent chitosan (Table 1). Free-base chitosan powders were dissolved at 20 mg/mL in dilute HCl overnight, then autoclave sterilized and characterized for solution pH, osmolality, and chitosan molecular weight (M_*w*_*)*[16] (Table 1). Chitosan *M*_w_, *M*_n_ and polydispersity index (PDI, *M*_w_/*M*_n_) was determined by size-exclusion chromatography with a Gel Permeation Chromatography System, coupled with a Dawn HELEOS II multiangle laser light scattering detector (Wyatt Technology Co., Santa Barbara, CA, USA), a Viscostar II (Wyatt Technology Co., Santa Barbara, CA, USA), an Optilab rEX interferometric refractometer (Wyatt Technology Co., Santa Barbara, CA, USA), and two Shodex OHpak columns (SB-806M HQ and SB-805 HQ) connected in series, as previously described [19]. Chitosan was dissolved at 1 mg/mL in running buffer (pH 4.5 acetic acid, 0.15 M/sodium acetate, 0.1 M) and filtered through a 0.45 μm membrane prior to the analysis. Rhodamine B isothiocyanate (RITC) labelled chitosans were generated from 3 structurally matched chitosans as described [16], precipitated in free-base form, dissolved in dilute HCl at 5 mg/mL and 90% protonation, 0.22 μm filter-sterilized, and the derivatization level determined by OD_540_ against a standard curve of rhodamine B (Table 1). All liquid chitosan aliquots were stored at −80°C and thawed once prior to use.

**Table 1:** Chitosans and chitosan solutions used in this study

**Chitosan**	**DDA (%)**	***M***_**w **_**(kDa)**	***M***_**n **_**(kDa)**	**PDI**	**Characteristics**
High (150K) Powder*	81.8	274.6	241.2	1.1	
High (150K) Solution**	81.8	124.2	107.4	1.2	pH=3.10 Osm=48mosm
RITC-150K ***	80.6	246.4	176	1.4	0.50% mol/mol RITC/chi
Low (40K) Powder*	81.9	47.7	37.3	1.3	
Low (40K) Solution**	81.9	31.6	25.3	1.2	pH=1.65 Osm=72mosm
RITC-40K ***	80.6	61.5	46.7	1.3	0.52% mol/mol RITC/chi
Ultralow (10K) Powder*	81.9	22.8	10.3	2.2	
Ultralow (10K) Solution**	81.9	23.8	12.7	1.9	pH=5.58 Osm=48mosm
RITC-10K ***	80.0	22.4	12.4	1.8	0.58% mol/mol RITC/chi

* Chitosan powder used to make the 20 mg/mL autoclave-sterilized solution; ** Autoclaved 20 mg/mL chitosan solution; *** RITC-chitosan powder used to make the 5 mg/mL filter-sterilized solution; DDA: Degree of deacetylation; *M*_w_: Weight-average molecular weight; *M*_n_: Number-average molecular weight; PDI: Polydispersity index (*M*_w_/*M*_n_); Osm: Osmolality; chi: chitosan.

Recombinant human Tissue Factor with phospholipids (Innovin®, Dade-Behring, Cedarlane, Mississauga, ON, Canada) was reconstituted with 2 mL water for injection (Abbott Laboratories, Dallas, TX, USA) at 5-times the normal recommended concentration and stored at 4°C. Microman M100 tips and microman pipettors were from Gilson, Inc. (Middleton, MI, USA). Recombinant human Factor VIIa (rhFVIIa, Novo Nordisk, Copenhagen, Denmark) was reconstituted at 500 μg/mL in water for injection and stored as aliquots at −80°C.

### Implant optimization and rabbit surgical model

All procedures involving animals, including a specific protocol for experimental procedures with aged rabbits, were approved by institutional ethics review boards. For *in vitro* optimization studies, isotonic chitosan solutions [1.6% w/v chitosan-150 mM NaCl or 1.6% w/v chitosan-100 mM disodium β-glycerol phosphate (GP)] were made from distinct 2% w/v chitosan solutions and laced with 0.05% w/v RITC-chitosan of matching molecular mass (Table 1). Formulations were mixed with rabbit peripheral whole blood from anesthetised rabbits at a 1:3 or 1:6 v/v chitosan:blood ratio in flat-bottom 2.0 mL cryovials containing three sterile 0.39 g surgical steel mixing beads (Salem Specialty Ball Company, Canton, CT, USA), and shaken manually for 10 seconds. Liquid mixtures were solidified in glass tubes with an inner diameter of 2.0 mm at 37°C for 30 minutes or at room temperature for 5 to 30 minutes in plastic syringes or microman tips with the tapered end cut off using rhFVIIa or Tissue Factor to accelerate coagulation. The macroscopic properties of the solidified implants (shape, elasticity, serum extruded) were determined visually and by manual assessment. The distribution of fluorescent chitosan particles in implants pre-solidified in glass tubes was documented by epifluorescence microscopy. Implants with 1:6 v/v ratio chitosan-NaCl/blood were selected for further analysis *in vivo* based on a more rapid coagulation than chitosan-GP/blood, and homogeneous dispersion of chitosan particles.

Five skeletally aged New Zealand White rabbits (2.5 years old, 3 male, 2 female, mean 4.68±0.44 kg, Charles River, St-Constant, QC, Canada), were housed individually in cages at controlled room temperature and light cycle with *ad libitum* standard commercial rabbit chow and municipal tap water available, and acclimatized for at least 2 weeks prior to surgery. The rabbits were anaesthetized with ketamine-xylazine-buprenorphine, had their knees shaved and disinfected and were kept under anaesthesia with 3% isoflurane/8% oxygen. Small bilateral arthrotomies were used to generate three osteochondral drill holes to 2 mm target depth in each femoral trochlea using a 1.4 mm diameter round drill burr (Fine Sciences Tools, Foster City, CA, USA) and a hand-held high speed drill under irrigation with Ringer’s Lactated Saline. For each rabbit, 3 mixtures were generated in distinct flat-bottom cryovials as described above, using 125 μL of 150K, 40K or 10K chitosan solution (1.6% w/v chitosan/150 mM NaCl solution), 12.5 μL 5 mg/mL RITC-chitosan of matching molecular mass (see Table 1) and 0.75 mL aseptic rabbit peripheral arterial liquid whole blood. 15 μL of the liquid implant mixture was drawn into a M100 sterile microman tip with the tapered end cut off. 3 μL of Tissue Factor was deposited in a sterile petri dish and then drawn into the same tip by back-screwing the microman pipettor to stimulate coagulation and thus solidify the implant inside the plastic microman tip (5 minutes at room temperature). Solid implants were extruded onto a sterile petri with labels, then each was press-fit into the 3 drill holes of the left knee (150K proximal, 40K middle, and 10K distal hole), while right knee control drill holes were left to bleed (N=2) or treated with 3 μL of Tissue Factor (3 μL per hole, N=2). Knees were closed in three layers using prolene 5–0 sutures, and buprenorphine was administered post-operatively twice a day for at least 3 days. Macroscopic appearance of the knees and incisions, body weight and signs of both pain and anorexia were monitored daily from the surgery to the necropsy to assess the health of the rabbits. There were no signs of knee infection at necropsy. After 1 day (N=1) or 21 days (N=4) post-surgery, the rabbits were euthanized under ketamine-xylazine anesthesia by IV injection of sodium pentobarbital and the femoral ends were retrieved and fixed in 4% paraformaldehyde/100mM cacodylate, pH 7.4. Fixed femur ends were placed trochlea face-down in buffered saline in a petri to acquire macroscopic fluorescent images of the defects with an inverted epifluorescent microscope, digital camera and calibrated histomorphometric software with a controlled image acquisition time (Northern Eclipse, Empix, Missisauga, ON, Canada).

### Micro-computed tomography (Micro-CT)

Femur ends were trimmed of their condyles using a low-speed isomet saw and scanned on a 180° axis using a SkyScan1172 high-resolution desktop X-ray microtomograph (Skyscan, Kontich, Belgium) set to an image size of 2000 X 1048 pixels at pixel size resolution of 9.8 μm, 2-frame averaging and at 80 kV with an aluminum-copper filter. The data sets were reconstructed in a 3D model using the NRecon software (Skyscan, Kontich, Belgium) with smoothing = 2, ring artifact correction = 4, beam hardening correction = 30%, object bigger than field of view = ON, output range = 0.00 to 0.05. The reconstructed files were blinded by a third party. Using the DataViewer software (Skyscan, Kontich, Belgium), each drill hole was re-positioned so that the y-axis ran vertically through the hole depth. The correct vertical alignment was then confirmed by two readers and the repositioned dataset was cropped into a new file. The top of each hole represented the osteochondral junction, visualized in corresponding histological sections as the tidemark of the calcified cartilage layer, and was defined as the most superficial image slice on the aligned reconstructed scans on which the bone completely surrounded the residual drill hole edges. The bottom of the hole was defined as the deepest axial slice that contained non-mineralized tissue, while a constant and dense bone zone could be observed at the base of the hole on both sagittal and coronal planes. The hole depth was calculated as the number of 2-D slices between the top and the bottom of the hole and multiplied by the pixel size (9.8 μm per slice). The residual hole cross-sectional area (mm^2^) was quantified at the top of the hole and at three different depths (0.5 mm, 1.0 mm and 1.5 mm) from the top of the hole using a polygon tool (CTAn software, Skyscan, Kontich, Belgium) to draw a 2D region of interest in the axial plane that circumscribed the non-mineralized hole edge (Additional file 1: Figure S1A). The signal inside the region of interest was then processed using an inverted 70–0 grayscale threshold to exclude mineralized tissue and include the non-mineralized drill hole cross-sectional area (Additional file 1: Figure S1B, C), which was then computed by the CTAn software.

### Histoprocessing, histostaining and immunohistochemistry

Femoral ends were decalcified at 4°C in 10% (w/v) ethylene diamine tetraacetic acid (EDTA)/0.1% (w/v) paraformaldehyde/phosphate buffered saline pH 7.2. 25 serial sagittal cryosections of 10 μm thickness were collected at the medial edge and through the middle of the 3 drill holes with CryoJane tape (Instrumedics, St Louis, MO, USA). Images of sections containing residual RITC-chitosan particles were generated with unstained and unmounted cryosections, by merging a 1.25x epifluorescence image with a low-intensity bright field image of the same field. Residual chitosan implant was measured by semiquantitative threshold analysis of red fluorescent particles in 1.25x fluorescent images acquired at a constant 400 ms exposure. Soft tissue in each hole was cropped into a new image, thresholded to a fixed value, and the total fluorescent area per hole was obtained in distinct sections from the edge and middle of the holes and the values averaged (N=4). Northern Eclipse software was used (Empix, Mississauga, ON, Canada). Cryosections were stained with Safranin O-fast green-iron hematoxylin, or immunostained for collagen type I (col I) and collagen type II (col II), following previously described methods [20], using anti-human collagen type I antibody I-8H5 (MP Biomedicals, Solon, OH, USA) at 10 μg/mL, anti-human collagen type II hybridoma supernatant II6B3 (DSHB, Iowa City, IA, USA) diluted 1:10 and biotinylated goat anti-mouse secondary antibody (B-7151; Sigma-Aldrich, Oakville, ON, Canada) at 11 μg/mL. Specificity was confirmed by the absence of immunostaining by isotype-specific mouse antisera or primary antibody omission.

Quantitative histomorphometry was carried out on calibrated 40x digitally scanned slides and Northern Eclipse V8.0 software (Empix Imaging, Inc., Mississauga, ON, Canada). Percent tissue stained for GAG, col I or col II was obtained by thresholding, and dividing by the total soft repair tissue area per drill hole. Histomorphometry of cross-sectional area (mm^2^) occupied by 4 granulation tissue types was carried out by selective cropping and threshold analysis of tissues from digital images of col I-immunostained sections (middle and edge of the holes), using the following criteria: (1) Apoptotic tissues contained mainly cells with fragmented nuclei, nuclear debris, and few cells with intact nuclei, (2) Neutrophil-rich tissues contained neutrophils with lobed or round nuclei, few stromal cells and low levels of apoptotic cells and nuclear debris, (3) Neutrophil+stromal tissues contained abundant neutrophils and stromal cells with an elongated fibroblast-like cell shape, (4) Stromal tissues were devoid of neutrophils and contained abundant stromal cells. Unbiased quantitative stereology for neutrophil and stromal cell density was carried out on a representative 40x image from each of the 4 tissue types in each treated defect (N=4, middle of the hole), over which a 20 x 20 μm grid, with 108 total points (P_total_), was randomly placed. All neutrophils or stromal cells touching the grid intersections were counted (Vv = ΣP_cell type_/ΣP_total_). A 100x picture of an apoptotic cell and a viable neutrophil were also taken. Repair tissue integration with the bone plate was analyzed in Safranin O, col I and col II-stained sections by calibrated histomorphometry. Line measures along the drill hole side-walls determined the distance between the osteochondral junction, as extrapolated from the tidemark in flanking cartilage-bone, and the closest subchondral repair tissue that was integrated to calcified cartilage or bone. Integration/detachment was measured on both sides of each hole and the mean value was retained. Cryosections were enzymatically stained with tartrate**-**resistant acidic phosphatase (TRAP) to reveal osteoclasts as previously described [10] and counterstained in methyl green (Vector Laboratories, Burlingame, CA, USA). TRAP-stained sections were digitally scanned at 40x. All large TRAP-positive cells adhering or adjacent to bone with a rounded cell shape were defined as osteoclasts [10] and were quantified in three different depths from the defect surface: projected tidemark-0.5 mm, 0.5 mm-1.0 mm and 1.0 mm-1.5mm, on both sides of each hole. The GAG, col I and col II-positive areas, as well as the osteoclast quantification, were performed by two independent blinded readers with consistent inter-reader results. Cryosections were also stained with Gomori Trichrome solution using 0.6% (w/v) Chromotrope 2R (C-3143, Sigma, Oakville, Ontario, Canada), 0.3% (w/v) Fast Green and 0.6% phosphotungstic acid (P-4006, Sigma, Oakville, Ontario, Canada).

### Statistical methods

The General Linear Model (GLM, Statistica version 9.0, StatSoft, Tulsa, OK, USA) with Fisher LSD post hoc analysis to analyze univariate effects was used to test the effect of chitosan molecular mass (150K, 40K, 10K) and defect position (proximal, mid, distal) after 21 days on chitosan residency, percent GAG-positive area, percent col I-positive area, percent col II-positive area, percent apoptotic, neutrophil-rich, neutrophil+stromal and stromal repair tissue area, neutrophil and stromal cell volume density, detached repair tissue, number of osteoclasts in three depth ranges, residual hole area at the top of the hole and at three depths and residual hole depth (N=4). Significance was set at p<0.05.

## Results

### *In vitro* pre-solidified chitosan/blood implant optimization

**Figure 1:**
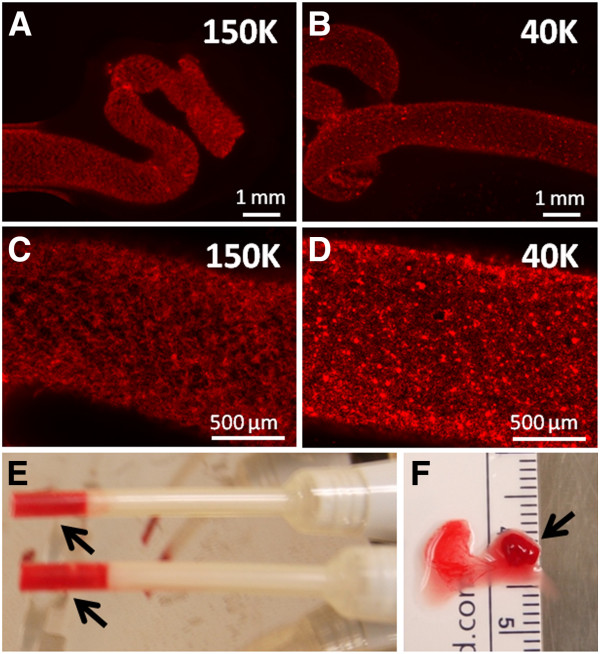
**Chitosan/blood implants form solid, cylindrical hybrid clots with punctate, insoluble RITC-chitosan particles distributed homogenously. (A, B)** 1.25x and **(C, D)** 5x magnification epifluorescence pictures of chitosan-GP/blood (1:6 v/v ratio) implants after solidification in glass tubes. **(E)** Solidification of the implants in M100 microman pipet tips (arrows) using Tissue Factor, and **(F)** solid chitosan-NaCl/blood implant (arrow), with some extruded serum.

Optimization studies were carried out *in vitro* to select a method to generate implants with homogeneously distributed chitosan particles that coagulated rapidly, which could facilitate their use in a clinical setting. Sterile chitosan solutions were successfully generated with high, low, and ultra-low molecular mass chitosans, here termed 150K, 40K, and 10K respectively (Table 1). Isotonic chitosan/blood mixtures formed solid, cylindrical elastic clots when incubated at 37°C in glass tubes, but remained liquid when incubated in plastic tubes (unpublished observations). These data were consistent with previous studies showing that chitosan-GP/blood mixtures solidify *via* normal coagulation mechanisms involving platelet and thrombin activation [11,21]. Pre-solidified implants showed a homogeneous punctate distribution of insoluble RITC-chitosan particles for 150K (Figure 1A, C), 40K (Figure 1B, D) and 10K (data not shown). Coagulation in plastic tubes at room temperature was enabled by addition of recombinant human Factor VIIa (rhFVIIa) to rabbit whole blood prior to mixing with chitosan (unpublished observations), or addition of a small volume of Tissue Factor (TF, Figure 1E). TF-chitosan-NaCl/blood implants formed rapidly (5 minutes) and retained a cylindrical shape after being extruded from the tip, with some serum expressed (Figure 1F).

### *In vivo* implant residency, repair responses and tissue characterization

**Figure 2:**
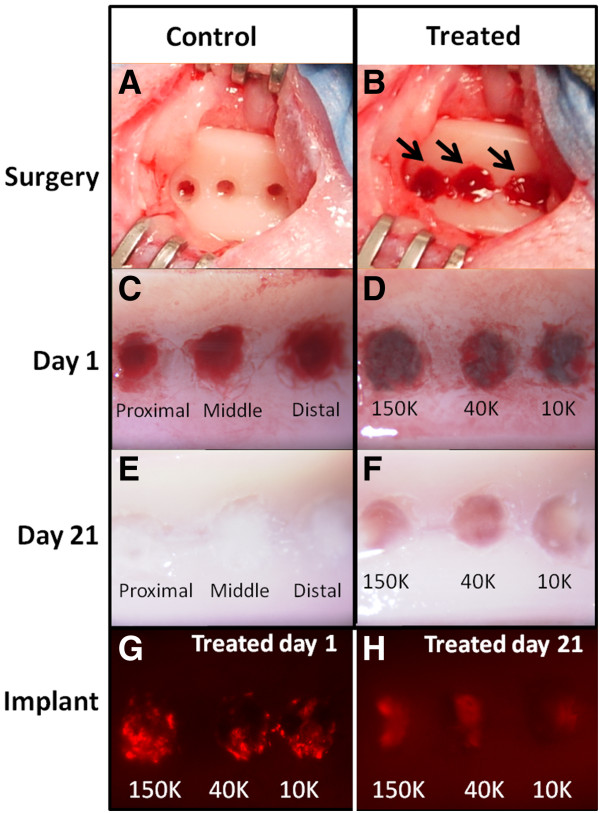
**Chitosan-NaCl/blood implants reside in osteochondral drill holes for 21 days post-operative. (A)** Microdrilled defects in rabbit trochlea. **(B)** Microdrilled defects in rabbit trochlea after press-fitting 3 pre-solidified implants (arrows). **(C)** Control defects after 1 day show a blood clot inside the holes (the distal control defect was treated with Tissue Factor). **(D)** Treated defects after 1 day show implants inside the holes. **(E)** Control defects after 3 weeks are filled with a white fibrous or fibrocartilage repair tissue. **(F)** Treated defects after 3 weeks are filled with a mixture of red angiogenic granulation tissue and white granulation tissue containing neutrophils or stromal cells. **(G, H)** 1.25x magnification epifluorescence pictures with 200ms exposure of whole knee trochlea shows three defects that retained RITC-chitosan after **(G)** 1 day and **(H)** 21 days.

Control drill defects were created in the femoral trochlea and allowed to fill with trabecular bone-derived blood, or treated with TF as a controlled variable (Figure 2A). Treated drill defects were press-fit with each of three distinct TF-chitosan-NaCl/blood implants with fluorescent chitosan tracer (150K, 40K, 10K) (Figure 2B). At 1 day post-operative, the microdrill holes were either filled with blood clot (Figure 2C) or implant (Figure 2D). At 21 days post-operative, all control defects were filled with a white repair tissue, while treated defects contained a mixture of white and red granulation tissue (Figure 2E, F). Macroscopically, RITC-chitosan was observed to be confined to the drill holes after both 1 and 21 days, with a fainter signal at 21 days (Figure 2G, H).

**Figure 3:**
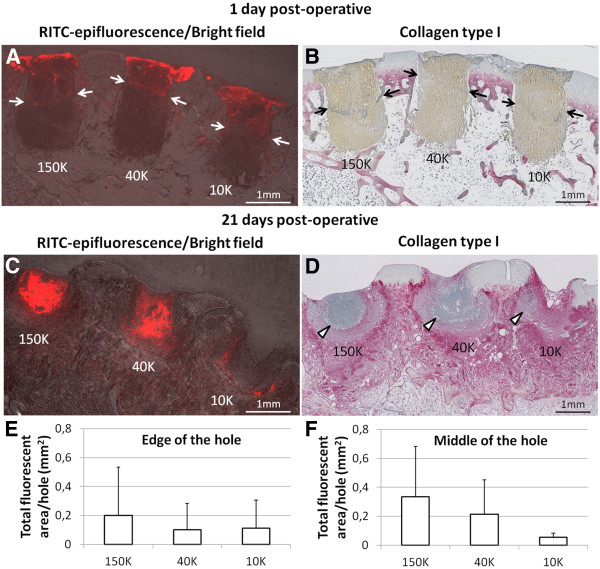
**Chitosan/blood implants reside near the top of the drill holes and elicit neutrophil chemotaxis. (A, C)** 1.25x magnification epifluorescence pictures of unstained sagittal cryosections taken in the middle of the holes, merged with bright field pictures to show the tissues. **(C, D)** 1.25x magnification pictures of collagen type I-immunostained serial sagittal sections adjacent to those shown in **(A)** and **(B)**. Panel **(A)** shows that RITC-chitosan particles in all 3 implants at day 1 are laterally filling the top half of the defects (red stain above white arrows). Panel **(C)** shows that RITC-chitosan particles persist with residual implant after 21 days *in vivo* and co-localize with areas of high-density neutrophils, as shown in **(D)**. **(E, F)** Total RITC-fluorescent area of each treated hole at 21 days post-operative (N=4) as taken from 1.25x magnification epifluorescence pictures of unstained cryosections taken at the edge of the hole **(E)** or in the middle of the hole **(F)**. (mean ± standard deviation). In the middle of the hole, 150K chitosan particles tended to reside longer in the defects than 10K chitosan particles (p=0.12, **F**).

**Figure 4:**
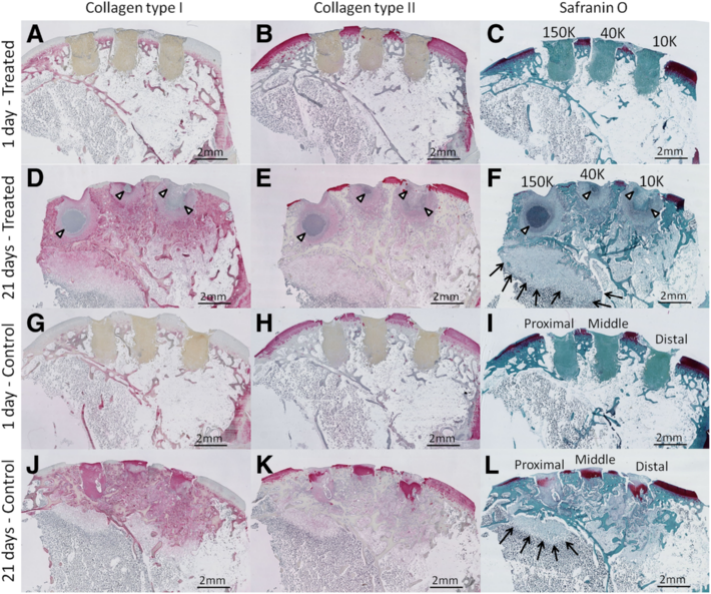
**Treated defects exhibit delayed collagen matrix deposition and strong neutrophil chemotaxis.** Representative 1.25x magnification pictures from serial sagittal sections taken in the middle of the holes in the same rabbit knee showing treated defects after **(A-C)** 1 day (N=1) and **(D-F)** 21 days (N=4), and control defects after **(G-I)** 1 day (N=1) and **(J-L)** 21 days (N=4). Panels show collagen type I-positive, collagen type II-positive, and Safranin O-stained glycosaminoglycan tissues. White arrowheads: neutrophil-rich granulation tissue, black arrows: local fibroplasia in bone marrow below the proximal drill holes.

Fluorescent chitosan particles underwent a dynamic change in distribution in the repairing bone defects over 21 days of repair. At day 1, chitosan implants laterally filled the upper portion of the drill holes (white arrows, Figure 3A) and contained scarce leukocytes within densely packed erythrocytes (Figure 3B). At 21 days post-operative, residual chitosan particles were dispersed unevenly throughout the holes and infiltrated with many neutrophils (Figure 3C-F). High molecular weight chitosan tended to clear more slowly from the defects compared to low molecular weight chitosan, as evidenced by the larger area of residual 150K chitosan compared to 10K chitosan in the middle of the holes (p=0.12, N=4, Figure 3F). Persistence of chitosan in all drill holes was associated with neutrophil chemotaxis, and delayed accumulation of col I, col II and glycosaminoglycans (GAG) in the soft repair tissues (Figure 4A-F).

**Figure 5:**
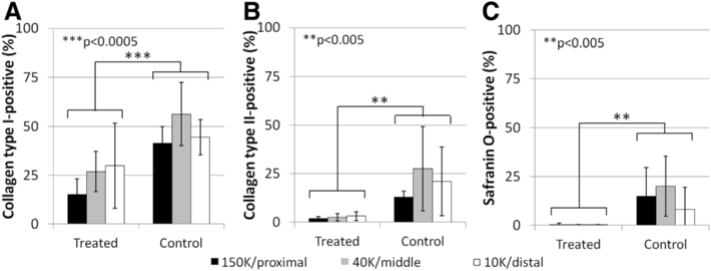
**Treated defects 21 days post-treatment contain less GAG, col II and col I than controls.** Percent of the soft repair tissue present in each drill hole cross-section in cryosections taken in the middle of the holes containing **(A)** collagen type I, **(B)** collagen type II, and **(C)** Safranin O-stained glycosaminoglycan, at 21 days (N=4) post-treatment. (mean ± standard deviation). **p<0.005, ***p<0.0005.

At 3 weeks post-operative, treated defects were mainly composed of granulation tissue (Figure 4D-F), whereas the blood clot initially formed in control bone defects was replaced by a variable mixture of GAG-depleted col I+/col II+ chondral repair tissues and GAG-rich, col I+/col II+ subchondral repair tissues (Figure 4G-L). Soft repair tissues formed in treated drill holes contained significantly less collagen type I (p<0.001), collagen type II (p<0.01) and GAG (p<0.01) than matching control defects (N=4; Figure 5).

**Figure 6:**
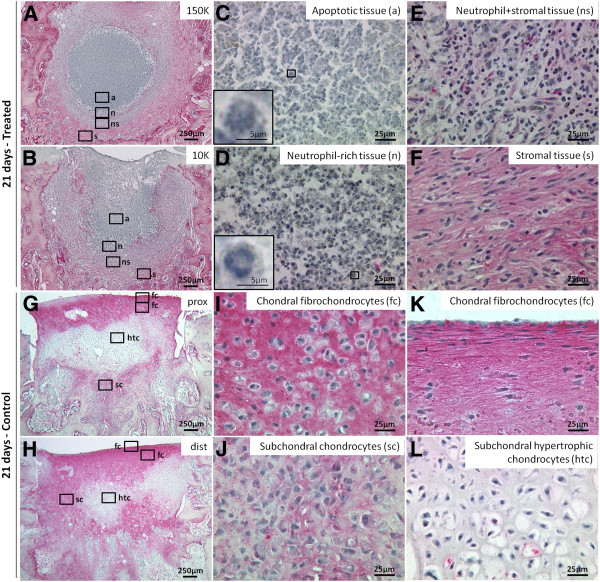
**Granulation tissues in treated defects *versus *chondral/subchondral tissues in control defects at 21 days post-operative**. Panels show collagen type I-immunostained/iron hematoxylin counterstained histology images at 1.25x **(A-B, G-H)** and 40x magnification **(C-F, I-L)**. **(A-F)** Treated repair tissues from representative 150K and 10K-treated defects contained apoptotic (a), neutrophil-rich (n), neutrophil+stromal (ns) and stromal (s) tissues. Control tissues from representative proximal and distal defects contained fibrochondrocytes (fc) above the osteochondral junction **(I, K)**, and subchondral chondrocytes (sc) or hypertrophic chondrocytes (htc) below the osteochondral junction **(J, L)**. At the articulating surface of the control defects, fibrochondrocytes and are identified by strong collagen type 1 expression and flattened morphology.

**Figure 7:**
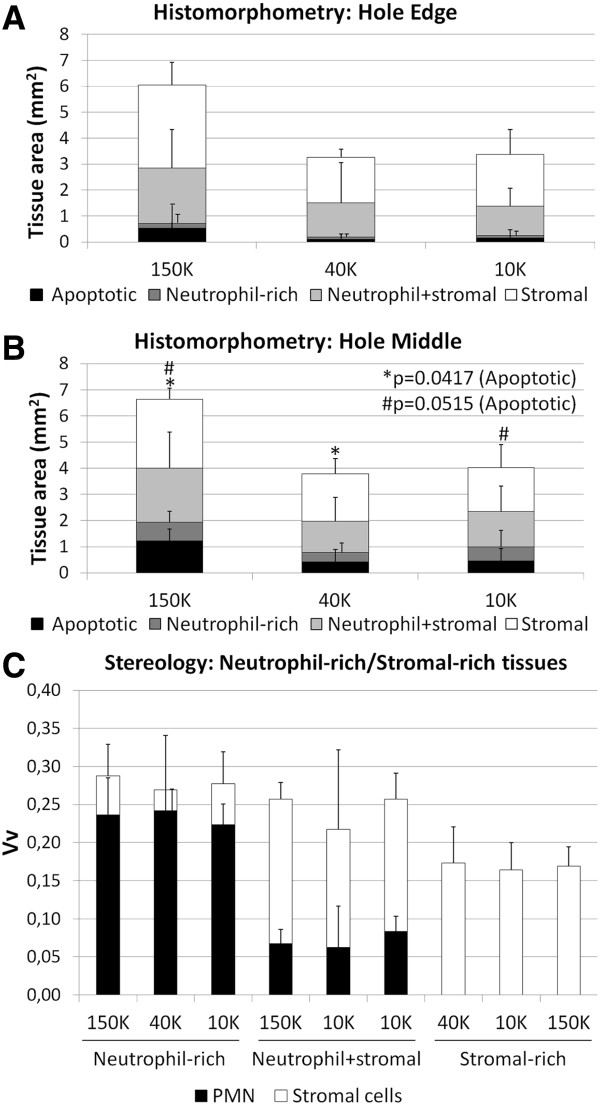
**Distribution of repair tissues in treated defects and volume density of neutrophils and stromal cells.** Relative cross-sectional area of apoptotic, neutrophil-rich, neutrophil+stromal and stromal tissues in sections collected **(A)** at the edge and **(B)** through the middle of treated defects in collagen type I-stained cryosections (N=4). Apoptotic tissues cover a larger area in the middle of 150K-treated defects after 21 days than 40K and 10K-treated defects (p=0.0417, p=0.0515, respectively). **(C)** Volume density (Vv) of neutrophils and stromal cells in 40x magnification pictures of neutrophil-rich, neutrophil+stromal and stromal tissues in the middle of day 21 treated defects of collagen type I-stained cryosections (N=4).

Four types of granulation tissue were observed in treated defects: apoptotic, neutrophil-rich, neutrophil+stromal and stromal (Figure 6A-F). A core of apoptotic tissue characterized the granulation tissue of 150K-treated defects, that covered a larger area than both 40K and 10K-treated defects (p=0.0417, p=0.0515, respectively, Figure 7A, B). From the periphery of apoptotic tissues to the edges of the holes, decreasing neutrophil counts and increasing levels of stromal cells were observed (Figure 7C). Control drill holes had a more advanced wound repair, and were mainly filled with collagen type I, interspersed with collagen type II and GAG (Figures 4–5). Above the osteochondral junction, control repair tissues contained numerous fibrochondrocytes and fibroblasts embedded in a collagen type I matrix (Figure 6I, K), while subchondral control tissues below the osteochondral junction were populated with fibrous tissue, chondrocytes and hypertrophic chondrocytes (Figure 6J, L). A local fibroplasia was also reproducibly observed in the metaphyseal marrow below the proximal control drill holes and proximal 150K-treated defects (Figure 4 F, L; black arrows).

**Figure 8:**
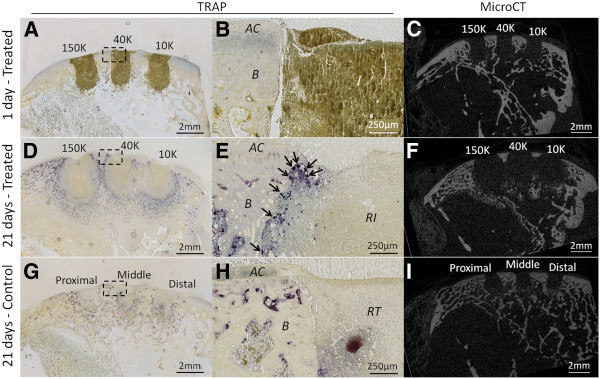
**Treated defects elicit osteoclast chemotaxis to the bone plate and subchondral bone remodeling.** Representative TRAP-stained decalcified sagittal sections at 1.25x **(A, D, G)** and 40x magnifications **(B, E, H)**. **(A, B)** No osteoclasts were present in day 1 defects. **(D, E)** In treated defects after 21 days, numerous osteoclasts have migrated to the subchondral bone plate. **(G, H)**. In control defects after 21 days, many osteoclasts have formed deeper in the repairing bone defect, but not at the subchondral bone plate level. **(C, F, I)** 2D-micro-CT images of mineralized tissue in matching sagittal sections show strong subchondral bone remodeling in treated defects, in contrast with the control defects with negligible remodeling of the hole sidewalls. *Symbols: AC*, Cartilage; *B*, Bone; *I*, Implant; *RI*, Residual implant; *RT*, Repair tissue.

**Figure 9:**
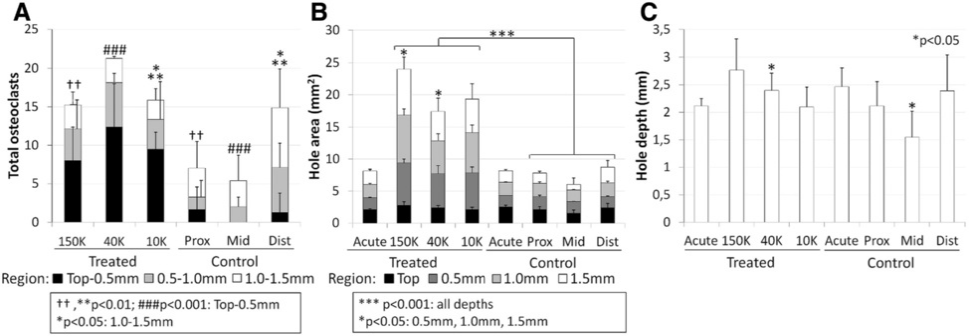
**Depth-wise measures of osteoclast chemotaxis and quantification of non-mineralized bone hole dimensions. (A)** Total number of TRAP+ cells lining both sides of each hole were quantified in three segments of each hole: the bone plate (tidemark-0.5mm deep), mid-level (0.5-1.0 mm deep) and deeper level (1.0-1.5mm deep). Compared to controls, treated defects showed more osteoclasts and osteoclast precursors in the bone plate region 21 days post-treatment (N=4). **(B)** Hole cross-sectional area at four depths (tidemark, 0.5 mm depth, 1.0 mm depth and 1.5 mm depth) in 3D microCT reconstructed scans in defects from 1 day (N=1) or 21 days (N=4) post-treatment. Treated drill holes became larger, and bone remodeling was particularly strong in 150K-treated defects at 0.5 mm and 1.5 mm depths compared to 40K-treated after 21 days. **(C)** Depth of the drill holes in micro-CT scans showed that middle control holes, after 21 days (N=4), were significantly more repaired at the base than the matching 40K-treated defects. (mean ± standard deviation). *p<0.05, **, ††p<0.01, ***, ###p<0.001.

Neutrophil migration to the implants in treated defects was accompanied by osteoclast chemotaxis to the bone plate region of all three chitosan formulations (Figure 8, Figure 9A). The bone plate region of treated drill holes (i.e., the tidemark to 0.5 mm deep), contained 10-fold more TRAP+ osteoclasts and osteoclast precursors on the defect edges compared to controls (Figure 8; p<0.01, N=4, Figure 9A). Deeper in the holes, treated defects contained similar or lower levels of TRAP+ osteoclasts and osteoclast precursors than controls (Figure 9A).

Initial defects by quantitative micro-CT analysis had a uniform ~1.6 mm diameter and 2.3 mm deep shape (N=6, Figure 9B-C). All implants induced bone resorption, which lead to tear drop-shaped bone defects at 3 weeks post-operative, as opposed to no appreciable change in control defect dimensions (Figure 9B-C). Compared to initial and control defects, all treated defects showed a ~2-fold increase in hole area at the calcified cartilage/bone plate (p<0.001), and ~3-fold wider hole area at deeper levels (p<0.001 *vs* controls, Figure 9B). By micro-CT, high molecular weight chitosan induced a particularly strong bone resorption at 0.5mm and 1.5mm depths (p<0.05, 150K *vs* 40K hole area, Figure 9B), which was consistent with histomorphometry measures in collagen type I-stained sections showing a larger area of granulation tissue in 150K-treated defects than in both 40K and 10K-treated defects after 21 days (p<0.05, data not shown). Micro-CT analysis of hole depth also showed that middle control bone defects were significantly shallower than the 40K-treated middle hole (p<0.05, Figure 9C).

**Figure 10:**
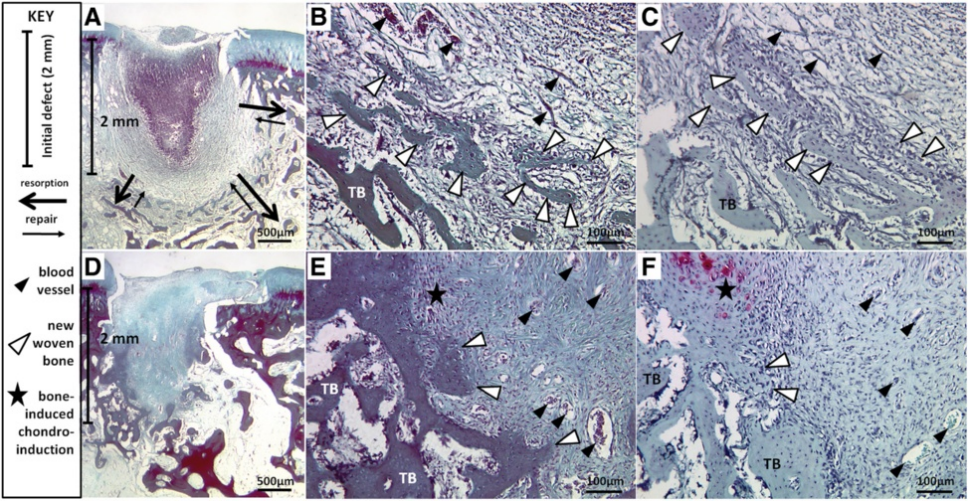
**Bone resorption and repair in chitosan-treated defects (A-C) *versus *minor endochondral bone repair in controls (D-F).** Gomori Trichrome-stained sagittal cryosections of **(A, B)** a 10K-treated defect and **(D, E)** a distal control defect and matching Safranin O-fast green-stained section **(C**, 10K-treated defect; **F**, control defect**)**. *Symbols:* Vertical bar: initial defect 2 mm depth; black arrows: zone of previous bone resorption (thick arrow) and woven bone repair (thin arrow); black arrowhead: blood vessels; white arrowhead: new woven bone; star: bone-induced chondroinduction [12,22].

The strong resorption in treated defects was accompanied by the synthesis of new woven bone and angiogenesis (Figure 10). Although the treated holes were bigger than the initial 2 mm deep, 1.4 mm diameter drill holes (Figure 9B, Figure 10A), subchondral new woven bone was identified by characteristic high-density osteocytes with a larger cell diameter compared to small osteocytes in peripheral non-remodeled trabecular bone (Figure 10B). Blood vessels were detected in the granulation tissue of treated defects (Figure 10B). The synthesis of new woven bone demonstrates that the implant has induced remodeling and not exclusively osteoclastic resorption. In control defects, no signs of bone resorption were present (Figure 9B, Figure 10D) and the subchondral bone contained large blood vessels, low levels of new woven bone, as chondrocytes fused to bone (Figure 10E) which indicates that endochondral ossification is underway.

**Figure 11:**
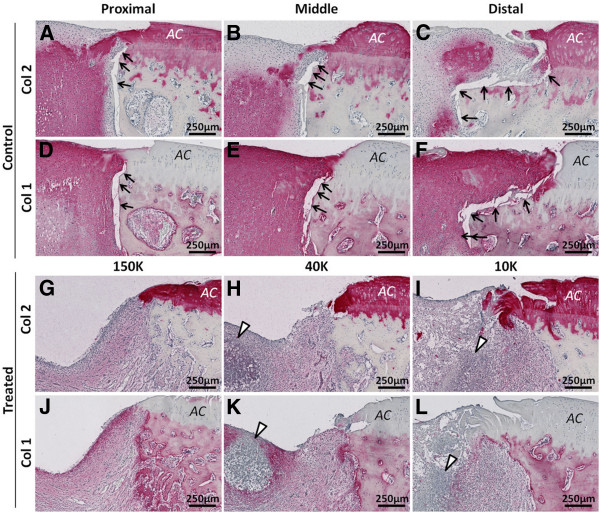
**Treated defects show better lateral repair tissue integration with the bone plate than control defects.** Panels **(A-C, G-I)** show collagen type II-stained sections and Panels **(D-F, J-L)** show collagen type I-stained sagittal sections from control defects **(A-F)** and treated defects **(G-L)** 21 days post-treatment). *Symbols: AC*: Articular cartilage; Black arrows: Detached repair tissue; White arrowheads: neutrophil-rich granulation tissue.

**Figure 12:**
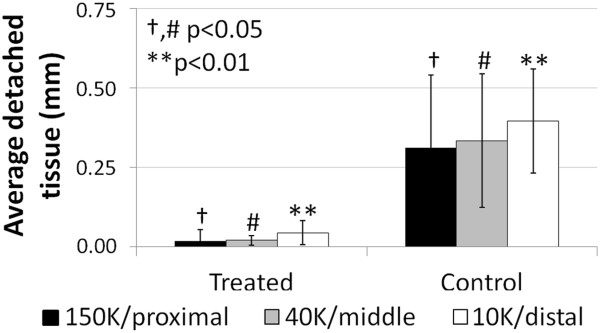
**Average detached repair tissue at the bone plate is greater in control than in treated defects.** Collagen-type II-stained sections in the middle of the holes, 21 days post-treatment. (mean ± standard deviation, N=4). #,†p<0.05. **p<0.01.

Osteoclast-induced bone remodeling in the bone plate area of treated defects was associated with a 10-fold greater lateral integration of treated repair tissues with the bone plate compared to control defects with detached repair (p<0.05, Figures 11 and 12). The mean lateral detachment was <35 μm deep in treated holes *versus* ~350 μm deep in control drill holes in sections immunostained for collagen type II (Figure 11A-C, Figure 12), collagen type I (Figure 11D-F), and stained for Safranin O (Figure 4L). Given that the bone plate (including the calcified cartilage) is ~400 μm thick in skeletally mature rabbits [23,24], these data showed that the marrow-derived fibrocartilage repair in control defects was generally not integrated with the bone plate. All three chitosan formulations reproducibly induced the same improved level of bone repair tissue integration.

## Discussion

This study demonstrated that subchondral chitosan implants sequentially draw neutrophils and undifferentiated bone marrow-derived stromal cells into drilled bone defects, which was accompanied by osteoclast formation and bone remodeling. Neutrophil attraction and bone resorption increased in proportion to higher chitosan molecular mass. After 3 weeks of repair, all chitosan implants induced neutrophil chemotaxis, stromal cell recruitment, delayed extracellular matrix deposition, and enhanced osteoclast-driven subchondral bone remodeling. Although both subchondral bone resorption and the synthesis of new woven bone were observed, the resorption was definitively stronger during the first three weeks of repair, as evidenced by the larger size of the treated holes compared to acute defects. At day 21, osteoclast recruitment below the bone plate was similar in both treated and control defects, which suggests a shift towards woven bone repair in treated defects after 21 days (Figure 10). Our data confirm the hypothesis that subchondral chitosan implants attract more bone marrow stromal cells than control defects in a time-delayed manner. Our data do not support the hypothesis that the implants are cleared with similar kinetics as the blood clot, given that chitosan particles still persisted at 3 weeks in the drill holes.

Biodegradable chitosan is known to elicit neutrophils *via* a leukotriene B4-sensitive pathway, while promoting an anti-inflammatory phenotype as chitosan-stimulated neutrophils release neither superoxide nor myeloperoxidase [25]. Previous chitosan-induced cartilage repair studies in rabbits have documented neutrophil chemotaxis at 21 days that is associated with a delay in matrix deposition, as observed in the current study [9,26]. The benefits of delayed matrix deposition after three weeks was also demonstrated in defects treated with PLG subchondral implants in rabbits, although implant clearance was not specifically reported and evidence of neutrophil or osteoclast attraction was not shown [3,27]. Most importantly, in a separate study, 10K pre-solidified chitosan-NaCl/blood implants elicited hyaline-like cartilage repair tissue and delayed callus formation compared to drill-only defects after 2.5 months in skeletally aged rabbits [28]. The more potent wound repair response to subchondral chitosan implants may therefore be the key to eliciting therapeutic marrow-stimulation repair tissues in aged subjects.

Although wound healing benefits from an early influx of neutrophils, failure to clear apoptotic neutrophils can cause impaired tissue repair [29,30], which could render the subchondral chitosan implant reactions problematic when scaling-up the implant to enhance marrow stimulation in patients. Efferocytosis, the uptake of apoptotic cells by macrophages [31], is a natural process in the resolution of inflammation. The low levels of apoptotic tissue in 10K and 40K-treated defects, as opposed to 150K-treated defects, suggest efficient efferocytosis by local macrophages that prevents triggering a foreign body response. The lower levels of residual apoptotic cells in the defects treated with low molecular weight chitosan (Figure 7B) correlate with sparse resident chitosan particles in the current study and a more rapid cell-based clearance of the smaller chitosan polymer chains, as previously documented [32]. Efferocytotic macrophages also secrete transforming growth factor-β [33,34], a key cytokine in wound healing [35], as well as the anti-inflammatory factor interleukin-10 [36], which further emphasizes the importance of proper neutrophil clearance in scaffold-guided osteochondral repair. While the areas in all defects that were characterized by high levels of neutrophils (viable or apoptotic) contained few stromal cells and no collagen type I, they were directly bordered by tissues rich with stromal cells (Vv_stromal_ = 0.17), at levels similar to that previously reported at 3 weeks post-operative in microdrill defects overlayed with chitosan-GP/blood implant (Vv_stromal_ = ~0.15) [9]. Stromal cells were thus recruited to the periphery of treated defects but only following timely neutrophil clearance; the sustained but more controlled neutrophil response for 40K and 10K implants therefore predicts a more therapeutic response at a later period in the rabbit model. This prediction is consistent with the outcome of a separate mid-term study in skeletally aged rabbits, in which 10K pre-solidified chitosan-NaCl/blood implants elicited a more hyaline repair tissue compared to 40K implant and untreated control drill holes [37].

Enhanced bone resorption correlated with the accumulation of neutrophils in treated defects compared to controls. The larger soft tissue area in 150K-treated defects also supports a relationship between prior neutrophil recruitment (i.e., apoptotic neutrophils present at 3 weeks in 150K-treated defects) and prior osteoclast activity. Receptor activator of NF-κB ligand (RANKL) induces the differentiation of osteoclast precursors [38,39]. In synovial fluid-derived neutrophils from patients with rheumatoid arthritis, as well as in blood-derived neutrophils stimulated with lipopolysaccharide and granulocyte macrophage colony-stimulating factor, RANKL is expressed and mediates bone resorption and osteoclastogenesis *in vitro*[40]. RANKL is also chemotactic for human monocytes [41,42]. Additionally, monocyte chemoattractant protein 1 (MCP-1/CCL2) and interleukin-8 (IL-8/CXCL8), are released from leukocytes within chitosan/blood clots and whole blood clots [26]. At the wound site, these chemokines could continue to be released from extravasated neutrophils [43] and endothelial cells through neutrophil mediators such as proteinase-3 [44,45]. In other studies, these chemokines stimulated osteoclast differentiation of human bone marrow mononuclear cells [46] and rat osteoclast precursor cells [47]. The local release of RANKL, MCP-1/CCL2 and IL-8/CXCL8 could thus polarize elicited macrophages to differentiate into osteoclasts in the drill hole. Interestingly, MCP-1/CCL2 could also have a role in neutrophil clearance, as it enhances efferocytosis [48]. Osteoclast formation promoted by “topical” chitosan-blood implants solidified over drill holes was previously tied to bone remodeling and improved repair tissue integration [10], as well as a more hyaline repair tissue at 8 weeks post-operative [9,11]. Osteoclast recruitment is also closely related to angiogenesis [49,50] and osteoclast conditioned media has a chemotactic effect on mesenchymal stem cells [51]. Osteoclast-dependant bone resorption could therefore enhance migration of mesenchymal stem cells to the bone plate region, which could be a key event in the cartilage repair properties of chitosan that is indirectly related to neutrophil recruitment. Stem cell recruitment could hold an especially important role in the treatment of cartilage defects in older patients who tend to be especially susceptible to microfracture failure [13,14]. Altogether, data in this study shows that pre-solidified chitosan-NaCl/blood implants can elicit osteoclasts to the subchondral bone plate, most probably through a neutrophil recruitment phase, and that molecular weight of the polymer can be used to adjust the potency of the bone remodeling response.

Development of chitosan-blood implants pre-solidified *ex vivo* provides a novel strategy for delivering specific dosages of low molecular weight chitosan (10K and 40K) with low viscosity [52] to marrow stimulation defects. Tissue Factor generated rapid implants which projects to be more convenient in a clinical setting, although Innovin® is not currently available as a pharmaceutical-grade reagent. Clinical use of Innovin® in dental sinus bone-augmentation procedures has been documented [53]. In this study, no apparent differences were observed between control defects left to bleed or administered with TF, although in a sheep model, subchondral TF implants were found to elicit sporadic bone plate resorption [Bell et al., unpublished data]. Future studies are needed to address the role of subchondral hemostasis and polymer clearance in marrow stimulation repair.

Chitosan-NaCl/blood pre-solidified subchondral implants elicited highly reproducible and distinct biological responses compared to controls. The statistically significant responses to implant compared to the controls is quite remarkable considering the low number of rabbits used (N=4). In the context of formulation screening, the current study also permitted the observation of greater neutrophil chemotaxis and bone resorption associated with slower clearance of 150K implants compared to 10K and 40K. These observations suggest that subchondral high molecular weight chitosan implants are at risk for the development of cysts at longer timepoints in the rabbit model, while low molecular weight chitosan implants have a greater potential for eliciting a delayed and improved osteochondral repair. The use of only one rabbit at day 1 was sufficient to document acute implant residency (Figures 2–3), and to document very uniform initial defect dimensions (Figure 9B-C), but did not permit any statistical comparison with day 21 defects. It is possible that proximo-distal effects on repair were present, as only the proximal holes showed fibroplasias in the deeper marrow. Such acute alterations in the deep marrow are likely to be transient given that adipocyte conversion in trabecular bone marrow is seen after 3 to 6 months post-operative below rabbit drilled defects [54,55]. The close proximity of the 3 drill holes in one trochlea may have biased the molecular mass-dependant repair reaction to the implants by those in the adjacent hole, although the study still permitted the observation of molecular weight-specific clearance and induction of neutrophil chemotaxis and bone resorption. These data have led us to change the model for mid-term repair responses by using only two drill holes per trochlea and further investigating the use of low molecular weight chitosan.

## Conclusions

This study has demonstrated that pre-solidified TF-chitosan-NaCl/blood implants can be successfully retained in microdrill holes, become partly cleared, and elicit neutrophils followed by bone marrow-derived stromal cells at 21 days in a skeletally aged rabbit model of cartilage repair. Three chitosan implant formulations reproducibly elicited neutrophils which guided osteoclasts to the bone plate, delayed deposition of collagen and glycosaminoglycan, induced subchondral bone resorption and repair and improved integration of the repair tissue with the subchondral bone. In contrast, in control defects, osteoclasts did not migrate to the bone plate, bone remodeling was limited to new woven bone at the base of the hole and stromal cells that migrated into the control drill holes promptly differentiated into chondrocytes and fibroblasts (col I-rich fibrocartilage). This study also showed that the level of neutrophil chemotaxis and osteoclast resorption was proportional to chitosan molecular weight. The successful characterization of the early, molecular weight-specific repair responses to osteochondral implants in aged rabbits constitutes a foundation for the elucidation of the molecular and cellular mechanisms of chitosan-guided neutrophil, stromal cell and osteoclast recruitment to marrow stimulation defects, as well as the further investigation of the cartilage repair properties of these implants at longer timepoints.

## Abbreviations

NaCl: Sodium chloride; DDA: Degree of deacetylation; 10K: 10 kilodaltons; 40K: 40 kilodaltons; 150K: 150 kilodaltons; Mw: Molecular weight; Mn: Number-average molecular weight; PDI: Polydispersity index; RITC: Rhodamine B isothiocyanate; TF: Tissue Factor; rhFVIIa: Recombinant human Factor VIIa; GP: β-glycerol phosphate; micro-CT: Micro-computed tomography; EDTA: Ethylene diamine tetraacetic acid; GAG: Glycosaminoglycan; col I: Collagen type I; col II: Collagen type II; Vv: Volume density; P: Point; TRAP: Tartrate**-**resistant Acidic Phosphatase; RANKL: Receptor activator of NF-κB ligand; MCP-1/CCL2: Monocyte chemoattractant protein 1 IL-8/CXCL8; IL-8/CXCL8: Interleukin-8; PLG: Poly(D,L-lactide-co-glycolide)

## Competing interests

This project was made possible by a grant from the Natural Sciences and Engineering Research Council of Canada SPG program. Some data in this paper were used towards a patent filing (WO2011060554-A1, CDH, CHLF, JS, JGM). None of the other authors has any competing interests to declare. All authors have had full access to the data and approve the final manuscript.

## Author’s contributions

CDH, WDS and JH designed the study; AH, TDH, JS and CDH optimized the *in vitro* implants; AH, TDH, JS and CDH performed the surgeries and collected safety data; JS prepared the chitosan solutions; CHLF, JGM, AH, TDH, JS and CDH performed the necropsies and collected the samples; CHLF, JGM and AC generated and analyzed micro-CT data; CHLF generated and quantified epifluorescence pictures; JGM, GC and CHLF did the histological and immunohistochemical stains; CHLF and GC performed histomorphometric and stereological analyses of histological sections; CHLF and CDH compiled data and performed the statistical analyses; CHLF generated the figures and wrote the manuscript; CDH, CHLF and JGM revised the manuscript. All authors approved the final manuscript.

## Pre-publication history

The pre-publication history for this paper can be accessed here:

http://www.biomedcentral.com/1471-2474/14/27/prepub

## Supplementary Material

Additional file 1: Figure S1 Micro-CT inverted thresholding method for quantification of residual (unrepaired) subchondral drill hole cross-sectional area. (A, B) An axial 2-D image of a residual drill hole in a reconstructed micro-CT data set in which a region of interest (excludes green area, includes black/white area) was drawn at the edge of the hole. (A) Original 70–255 thresholded binary image (white: bone, black: non-mineralized tissue) and (B) inverted 70–0 thresholded binary image (white: non-mineralized tissue, black: bone). (C) After cropping with the inverted 70–0 threshold, the residual hole cross-sectional area inside the region of interest is obtained through the calibrated micro-CT software. Click here for file

## References

[B1] Breinan HA Martin SD Hsu HP Spector M Healing of canine articular cartilage defects treated with microfracture, a type-II collagen matrix, or cultured autologous chondrocytes J Orthop Res 2000 18 781 789 10.1002/jor.1100180516 11117301

[B2] Knutsen G Drogset JO Engebretsen L Grontvedt T Isaksen V Ludvigsen TC Roberts S Solheim E Strand T Johansen O A randomized trial comparing autologous chondrocyte implantation with microfracture. Findings at five years J Bone Joint Surg Am 2007 89 2105 2112 10.2106/JBJS.G.00003 17908884

[B3] Toyokawa N Fujioka H Kokubu T Nagura I Inui A Sakata R Satake M Kaneko H Kurosaka M Electrospun synthetic polymer scaffold for cartilage repair without cultured cells in an animal model Arthroscopy 2010 26 375 383 10.1016/j.arthro.2009.08.006 20206048

[B4] Carmont MR Carey-Smith R Saithna A Dhillon M Thompson P Spalding T Delayed incorporation of a TruFit plug: perseverance is recommended Arthroscopy 2009 25 810 814 10.1016/j.arthro.2009.01.023 19560648

[B5] Barber FA Dockery WD A computed tomography scan assessment of synthetic multiphase polymer scaffolds used for osteochondral defect repair Arthroscopy 2011 27 60 64 10.1016/j.arthro.2010.06.023 20952149

[B6] Streitparth F Schottle P Schlichting K Schell H Fischbach F Denecke T Duda GN Schroder RJ Osteochondral defect repair after implantation of biodegradable scaffolds: indirect magnetic resonance arthrography and histopathologic correlation Acta Radiol 2009 50 765 774 10.1080/02841850902980272 19626474

[B7] Hoemann CD Sun J Legare A McKee MD Buschmann MD Tissue engineering of cartilage using an injectable and adhesive chitosan-based cell-delivery vehicle Osteoarthr Cartil 2005 13 318 329 10.1016/j.joca.2004.12.001 15780645

[B8] Hoemann CD Hurtig M Rossomacha E Sun J Chevrier A Shive MS Buschmann MD Chitosan-glycerol phosphate/blood implants improve hyaline cartilage repair in ovine microfracture defects J Bone Joint Surg Am 2005 87 2671 2686 10.2106/JBJS.D.02536 16322617

[B9] Chevrier A Hoemann CD Sun J Buschmann MD Chitosan-glycerol phosphate/blood implants increase cell recruitment, transient vascularization and subchondral bone remodeling in drilled cartilage defects Osteoarthr Cartil 2007 15 316 327 10.1016/j.joca.2006.08.007 17008111

[B10] Chen G Sun J Lascau-Coman V Chevrier A Marchand C Hoemann CD Acute Osteoclast Activity following Subchondral Drilling Is Promoted by Chitosan and Associated with Improved Cartilage Repair Tissue Integration Cartilage 2011 2 173 185 10.1177/1947603510381096 PMC4300782 26069578

[B11] Hoemann CD Sun J McKee MD Chevrier A Rossomacha E Rivard GE Hurtig M Buschmann MD Chitosan-glycerol phosphate/blood implants elicit hyaline cartilage repair integrated with porous subchondral bone in microdrilled rabbit defects Osteoarthr Cartil 2007 15 78 89 10.1016/j.joca.2006.06.015 16895758

[B12] Chevrier A Hoemann CD Sun J Buschmann MD Temporal and spatial modulation of chondrogenic foci in subchondral microdrill holes by chitosan-glycerol phosphate/blood implants Osteoarthr Cartil 2011 19 136 144 10.1016/j.joca.2010.10.026 21044693

[B13] Kreuz PC Erggelet C Steinwachs MR Krause SJ Lahm A Niemeyer P Ghanem N Uhl M Sudkamp N Is microfracture of chondral defects in the knee associated with different results in patients aged 40 years or younger? Arthroscopy 2006 22 1180 1186 10.1016/j.arthro.2006.06.020 17084294

[B14] Mithoefer K Williams RJ Warren RF Potter HG Spock CR Jones EC Wickiewicz TL Marx RG The microfracture technique for the treatment of articular cartilage lesions in the knee. A prospective cohort study J Bone Joint Surg Am 2005 87 1911 1920 10.2106/JBJS.D.02846 16140804

[B15] Chen H Sun J Hoemann CD Lascau-Coman V Ouyang W McKee MD Shive MS Buschmann MD Drilling and microfracture lead to different bone structure and necrosis during bone-marrow stimulation for cartilage repair J Orthop Res 2009 27 1432 1438 10.1002/jor.20905 19402150

[B16] Ma O Lavertu M Sun J Nguyen S Buschmann MD Winnik FM Hoemann CD Precise derivatization of structurally distinct chitosans with rhodamine B isothiocyanate Carbohydr Polym 2008 72 616 624 10.1016/j.carbpol.2007.10.004

[B17] Lavertu M Methot S Tran-Khanh N Buschmann MD High efficiency gene transfer using chitosan/DNA nanoparticles with specific combinations of molecular weight and degree of deacetylation Biomaterials 2006 27 4815 4824 10.1016/j.biomaterials.2006.04.029 16725196

[B18] Allan GG Peyron M **Molecular weight manipulation of chitosan.** I: Kinetics of depolymerization by nitrous acid Carbohydr Res 1995 277 257 272 10.1016/0008-6215(95)00207-A 8556735

[B19] Nguyen S Winnik FM Buschmann MD Improved reproducibility in the determination of the molecular weight of chitosan by analytical size exclusion chromatography Carbohydr Polym 2009 75 528 533 10.1016/j.carbpol.2008.08.013

[B20] Chevrier A Rossomacha E Buschmann MD Hoemann CD Optimization of histoprocessing methods to detect glycosaminoglycan, collagen type II, and collagen type I in decalcified rabbit osteochondral sections J Histotechnology 2005 28 165 175 10.1179/014788805794775145

[B21] Marchand C Rivard GE Sun J Hoemann CD Solidification mechanisms of chitosan-glycerol phosphate/blood implant for articular cartilage repair Osteoarthr Cartil 2009 17 953 960 10.1016/j.joca.2008.12.002 19152788

[B22] Bell AD Lascau-Coman V Sun J Chen G Lowerison MW Hurtig MB Hoemann CD Bone-induced chondroinduction in sheep jamshidi biopsy defects with and without treatment by Subchondral Chitosan-Blood Implant: 1-day, 3-week, and 3-month repair Cartilage 2012 e-pub ahead of print 10.1177/1947603512463227 PMC4297102 26069656

[B23] Marchand C Chen H Buschmann MD Hoemann CD Standardized three-dimensional volumes of interest with adapted surfaces for more precise subchondral bone analyses by micro-computed tomography Tissue Eng Part C Methods 2011 17 475 484 21142419 10.1089/ten.TEC.2010.0417

[B24] Frisbie DD Cross MW McIlwraith CW A comparative study of articular cartilage thickness in the stifle of animal species used in human pre-clinical studies compared to articular cartilage thickness in the human knee Vet Comp Orthop Traumatol 2006 19 142 146 16971996

[B25] Simard P Galarneau H Marois S Rusu D Hoemann CD Poubelle PE El-Gabalawy H Fernandes MJ Neutrophils exhibit distinct phenotypes toward chitosans with different degrees of deacetylation: implications for cartilage repair Arthritis Res Ther 2009 11 R74 10.1186/ar2703 19460141 PMC2714120

[B26] Hoemann CD Chen G Marchand C Tran-Khanh N Thibault M Chevrier A Sun J Shive MS Fernandes MJ Poubelle PE Scaffold-guided subchondral bone repair: implication of neutrophils and alternatively activated arginase-1+ macrophages Am J Sports Med 2010 38 1845 1856 10.1177/0363546510369547 20522834

[B27] Sakata R Kokubu T Nagura I Toyokawa N Inui A Fujioka H Kurosaka M Localization of vascular endothelial growth factor during the early stages of osteochondral regeneration using a bioabsorbable synthetic polymer scaffold J Orthop Res 2012 30 252 259 10.1002/jor.21502 21809378

[B28] Hoemann CD Lafantaisie-Favreau CH Lascau-Coman V Chen G Guzman-Morales J The cartilage-bone interface J Knee Surg 2012 25 85 97 22928426 10.1055/s-0032-1319782

[B29] Khanna S Biswas S Shang Y Collard E Azad A Kauh C Bhasker V Gordillo GM Sen CK Roy S Macrophage dysfunction impairs resolution of inflammation in the wounds of diabetic mice PLoS One 2010 5 e9539 10.1371/journal.pone.0009539 20209061 PMC2832020

[B30] Jonsson H Allen P Peng SL Inflammatory arthritis requires Foxo3a to prevent Fas ligand-induced neutrophil apoptosis Nat Med 2005 11 666 671 10.1038/nm1248 15895074

[B31] de Cathelineau AM Henson PM The final step in programmed cell death: phagocytes carry apoptotic cells to the grave Essays Biochem 2003 39 105 117 14585077 10.1042/bse0390105

[B32] Onishi H Machida Y Biodegradation and distribution of water-soluble chitosan in mice Biomaterials 1999 20 175 182 10.1016/S0142-9612(98)00159-8 10022787

[B33] Fadok VA Bratton DL Konowal A Freed PW Westcott JY Henson PM Macrophages that have ingested apoptotic cells in vitro inhibit proinflammatory cytokine production through autocrine/paracrine mechanisms involving TGF-beta, PGE2, and PAF J Clin Invest 1998 101 890 898 10.1172/JCI1112 9466984 PMC508637

[B34] Huynh ML Fadok VA Henson PM Phosphatidylserine-dependent ingestion of apoptotic cells promotes TGF-beta1 secretion and the resolution of inflammation J Clin Invest 2002 109 41 50 11781349 10.1172/JCI11638 PMC150814

[B35] Faler BJ Macsata RA Plummer D Mishra L Sidawy AN Transforming growth factor-beta and wound healing Perspect Vasc Surg Endovasc Ther 2006 18 55 62 10.1177/153100350601800123 16628336

[B36] Voll RE Herrmann M Roth EA Stach C Kalden JR Girkontaite I Immunosuppressive effects of apoptotic cells Nature 1997 390 350 351 10.1038/37022 9389474

[B37] Guzman-Morales J Lafantaisie-Favreau C-H Sun J Rivard GE Hoemann CD Analysis of the mid-term effects of chitosan-NaCl/blood pre-solidified implants in an in vivo osteochondral repair model9th World Congress of the International Cartilage Repair Society 2010 Sitges, Spain: Cartilage 74S 150S 1

[B38] Boyce BF Xing L Functions of RANKL/RANK/OPG in bone modeling and remodeling Arch Biochem Biophys 2008 473 139 146 10.1016/j.abb.2008.03.018 18395508 PMC2413418

[B39] Yamashita T Yao Z Li F Zhang Q Badell IR Schwarz EM Takeshita S Wagner EF Noda M Matsuo K NF-kappaB p50 and p52 regulate receptor activator of NF-kappaB ligand (RANKL) and tumor necrosis factor-induced osteoclast precursor differentiation by activating c-Fos and NFATc1 J Biol Chem 2007 282 18245 18253 10.1074/jbc.M610701200 17485464

[B40] Chakravarti A Raquil MA Tessier P Poubelle PE Surface RANKL of Toll-like receptor 4-stimulated human neutrophils activates osteoclastic bone resorption Blood 2009 114 1633 1644 10.1182/blood-2008-09-178301 19546479

[B41] Breuil V Schmid-Antomarchi H Schmid-Alliana A Rezzonico R Euller-Ziegler L Rossi B The receptor activator of nuclear factor (NF)-kappaB ligand (RANKL) is a new chemotactic factor for human monocytes FASEB J 2003 17 1751 1753 12958198 10.1096/fj.02-1188fje

[B42] Mosheimer BA Kaneider NC Feistritzer C Sturn DH Wiedermann CJ Expression and function of RANK in human monocyte chemotaxis Arthritis Rheum 2004 50 2309 2316 10.1002/art.20352 15248232

[B43] Yamashiro S Kamohara H Yoshimura T MCP-1 is selectively expressed in the late phase by cytokine-stimulated human neutrophils: TNF-alpha plays a role in maximal MCP-1 mRNA expression J Leukoc Biol 1999 65 671 679 10331497 10.1002/jlb.65.5.671

[B44] Taekema-Roelvink ME Kooten C Kooij SV Heemskerk E Daha MR Proteinase 3 enhances endothelial monocyte chemoattractant protein-1 production and induces increased adhesion of neutrophils to endothelial cells by upregulating intercellular cell adhesion molecule-1 J Am Soc Nephrol 2001 12 932 940 11316851 10.1681/ASN.V125932

[B45] Berger SP Seelen MA Hiemstra PS Gerritsma JS Heemskerk E van der Woude FJ Daha MR Proteinase 3, the major autoantigen of Wegener’s granulomatosis, enhances IL-8 production by endothelial cells in vitro J Am Soc Nephrol 1996 7 694 701 8738804 10.1681/ASN.V75694

[B46] Lu Y Cai Z Xiao G Keller ET Mizokami A Yao Z Roodman GD Zhang J Monocyte chemotactic protein-1 mediates prostate cancer-induced bone resorption Cancer Res 2007 67 3646 3653 10.1158/0008-5472.CAN-06-1210 17440076

[B47] Asano M Yamaguchi M Nakajima R Fujita S Utsunomiya T Yamamoto H Kasai K IL-8 and MCP-1 induced by excessive orthodontic force mediates odontoclastogenesis in periodontal tissues Oral Dis 2011 17 489 498 10.1111/j.1601-0825.2010.01780.x 21496183

[B48] Tanaka T Terada M Ariyoshi K Morimoto K Monocyte chemoattractant protein-1/CC chemokine ligand 2 enhances apoptotic cell removal by macrophages through Rac1 activation Biochem Biophys Res Commun 2010 399 677 682 10.1016/j.bbrc.2010.07.141 20691665

[B49] Cheung WY Liu C Tonelli-Zasarsky RM Simmons CA You L Osteocyte apoptosis is mechanically regulated and induces angiogenesis in vitro J Orthop Res 2011 29 523 530 10.1002/jor.21283 21337392

[B50] Cackowski FC Anderson JL Patrene KD Choksi RJ Shapiro SD Windle JJ Blair HC Roodman GD Osteoclasts are important for bone angiogenesis Blood 2010 115 140 149 10.1182/blood-2009-08-237628 19887675 PMC3988688

[B51] Kreja L Brenner RE Tautzenberger A Liedert A Friemert B Ehrnthaller C Huber-Lang M Ignatius A Non-resorbing osteoclasts induce migration and osteogenic differentiation of mesenchymal stem cells J Cell Biochem 2010 109 347 355 19950208 10.1002/jcb.22406

[B52] Kasaai MR Arul J Charlet G Intrinsic viscosity–molecular weight relationship for chitosan J Polym Sci Part B: Polym Phys 2000 38 2591 2598 10.1002/1099-0488(20001001)38:19<2591::AID-POLB110>3.0.CO;2-6

[B53] Philippart P Brasseur M Hoyaux D Pochet R Human recombinant tissue factor, platelet-rich plasma, and tetracycline induce a high-quality human bone graft: a 5-year survey Int J Oral Maxillofac Implants 2003 18 411 416 12814317

[B54] Chen H Hoemann CD Sun J Chevrier A McKee MD Shive MS Hurtig M Buschmann MD Depth of subchondral perforation influences the outcome of bone marrow stimulation cartilage repair J Orthop Res 2011 29 1178 1184 10.1002/jor.21386 21671261

[B55] Marchand C Chen G Tran-Khanh N Sun J Chen H Buschmann MD Hoemann CD Microdrilled cartilage defects treated with thrombin-solidified chitosan/blood implant regenerate a more hyaline, stable, and structurally integrated osteochondral unit compared to drilled controls Tissue Eng Part A 2012 18 508 519 10.1089/ten.tea.2011.0178 21942869

