# Supplementary figures and images for: Subchondral pre-solidified chitosan/blood implants elicit reproducible early osteochondral wound-repair responses including neutrophil and stromal cell chemotaxis, bone resorption and repair, enhanced repair tissue integration and delayed matrix deposition
Source: BMC Musculoskelet Disord. 2013 Jan 16;14:27. doi: 10.1186/1471-2474-14-27 (PMC3602124; doi:10.1186/1471-2474-14-27)

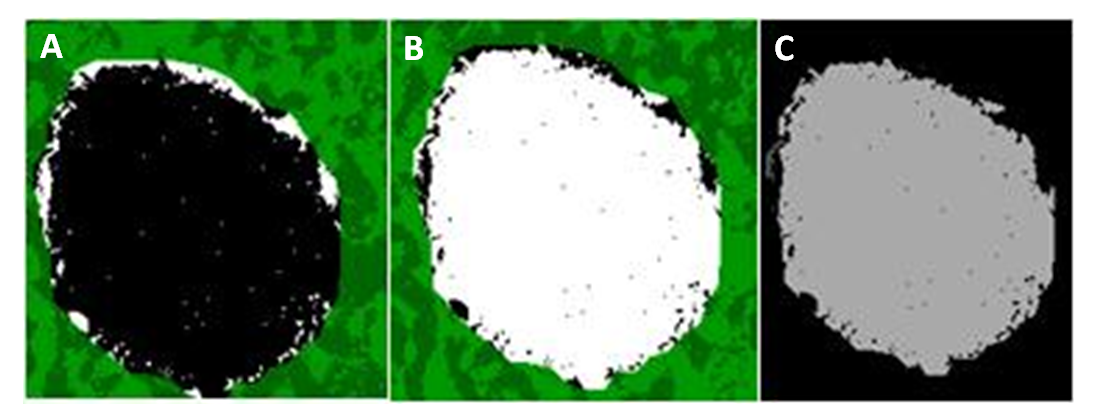

Supplement: Additional file 1: Figure S1 — Micro-CT inverted thresholding method for quantification of residual (unrepaired) subchondral drill hole cross-sectional area. (A, B) An axial 2-D image of a residual drill hole in a reconstructed micro-CT data set in which a region of interest (excludes green area, includes black/white area) was drawn at the edge of the hole. (A) Original 70–255 thresholded binary image (white: bone, black: non-mineralized tissue) and (B) inverted 70–0 thresholded binary image (white: non-mineralized tissue, black: bone). (C) After cropping with the inverted 70–0 threshold, the residual hole cross-sectional area inside the region of interest is obtained through the calibrated micro-CT software. [file 1471-2474-14-27-S1.tiff]
